# Anti-Inflammatory Effect of Targeted Delivery of SOD to Endothelium: Mechanism, Synergism with NO Donors and Protective Effects *In Vitro* and *In Vivo*

**DOI:** 10.1371/journal.pone.0077002

**Published:** 2013-10-11

**Authors:** Vladimir V. Shuvaev, Jingyan Han, Samira Tliba, Evguenia Arguiri, Melpo Christofidou-Solomidou, Servio H. Ramirez, Holly Dykstra, Yuri Persidsky, Dmitriy N. Atochin, Paul L. Huang, Vladimir R. Muzykantov

**Affiliations:** 1 Department of Pharmacology and Center for Translational Targeted Therapeutics and Nanomedicine of the Institute for Translational Medicine and Therapeutics, University of Pennsylvania, Philadelphia, Pennsylvania, United States of America.; 2 Department of Medicine, Pulmonary, Allergy and Critical Care Division, University of Pennsylvania, Philadelphia, Pennsylvania, United States of America; 3 Department of Pathology and Laboratory Medicine, Temple University School of Medicine, Philadelphia, Pennsylvania, United States of America; 4 Cardiovascular Research Center and Cardiology Division, Department of Medicine, Massachusetts General Hospital and Harvard Medical School, Boston, Massachusetts, United States of America; University of Illinois at Chicago, United States of America

## Abstract

Pro-inflammatory activation of vascular endothelium is implicated in pathogenesis of severe conditions including stroke, infarction and sepsis. We have recently reported that superoxide dismutase (SOD) conjugated with antibodies (Ab/SOD) that provide targeted delivery into endothelial endosomes mitigates inflammatory endothelial activation by cytokines and agonists of Toll-like receptors (TLR). The goal of this study was to appraise potential utility and define the mechanism of this effect. Ab/SOD, but not non-targeted SOD injected in mice alleviated endotoxin-induced leukocyte adhesion in the cerebral vasculature and protected brain from ischemia-reperfusion injury. Transfection of endothelial cells with SOD, but not catalase inhibited NFκB signaling and expression of Vascular Cell Adhesion Molecule-1 induced by both cytokines and TLR agonists. These results affirmed that Ab/SOD-quenched superoxide anion produced by endothelial cells in response to proinflammatory agents mediates NFκB activation. Furthermore, Ab/SOD potentiates anti-inflammatory effect of NO donors in endothelial cells in vitro, as well as in the endotoxin-challenged mice. These results demonstrate the central role of intracellular superoxide as a mediator of pro-inflammatory activation of endothelium and support the notion of utility of targeted interception of this signaling pathway for management of acute vascular inflammation.

## Introduction

Pro-inflammatory activation of vascular endothelium caused by ischemia, infectious agents, cytokines, reactive oxygen species (ROS) including superoxide and H_2_O_2_ and other pathological mediators, is implicated in cardiac, pulmonary, cerebral and peripheral vascular pathology [1], [2], [3], [4]. Such activation, manifested among other signs by expression of cell adhesion molecules including vascular cell adhesion molecule 1 (VCAM), in turn aggravates inflammation via adhesion and trafficking of activated leukocytes, enhanced vascular permeability and thrombosis [5]. Recent studies implicated ROS produced by vascular cells as both injurious agents and pro-inflammatory signaling mediators in vascular pathology [6]. In particular, ROS produced by NADPH oxidase in the lumen of endothelial endosomes in response to cytokines, are implicated in signaling for pro-inflammatory endothelial activation [7]. Interruption of this pathological pathway may provide a new means for alleviation of uncontrolled vascular inflammation implicated in the pathogenesis of devastating conditions including acute lung injury (ALI), stroke and myocardial infarction.

Results of our recent study suggest that this goal can be achieved by targeted delivery of antioxidant enzymes to endothelial cells [8]. Conjugation with antibodies to endothelial surface marker molecule platelet endothelial cell adhesion molecule 1 (PECAM) provides targeted delivery of antioxidant enzymes catalase and superoxide dismutase (SOD) into endothelial cells of lungs, heart and brain [9], [10], [11]. In particular, SOD conjugated with anti-PECAM (Ab/SOD) binds to and enters endothelial endosomes, quenches superoxide in endosomes and inhibits cytokine-induced pro-inflammatory VCAM expression induced by cytokines or agonists of Toll-like receptors (TLR) [8], [12]. Non-targeted SOD formulations including polyethylene glycol (PEG)-conjugated SOD, which have no affinity to endothelium and little, if any, access to intracellular superoxide, do not afford this effect despite a much higher level in the blood [8]. These findings unraveled a new paradigm for protective effects of targeted antioxidants, an area of active translational research that yielded encouraging pre-clinical results in animal models of oxidative stress, ischemia and inflammation [13]. In the present study, we investigated the mechanism and translational potential of the anti-inflammatory action of Ab/SOD *in vitro* and *in vivo*.

## Materials and Methods

### Reagents, Antibodies and Conjugates

Cytochrome c, xanthine oxidase, xanthine (3,7-dihydro-purine-2,6-dione), dimethylformamide (DMFA) and fetal bovine serum were purchased from Sigma (St. Louis, MO). Cu, Zn-superoxide dismutase (SOD) and catalase from bovine liver are from Calbiochem (San Diego, CA). Succinimidyl-6-[biotinamido]hexanoate (NHS-LC-biotin), 4-[*N*-maleimidomethyl]cyclohexane-1-carboxylate (SMCC), N-succinimidyl-S-acetylthioacetate (SATA) were from Pierce Biotechnology (Rockford, IL). Anti-PECAM monoclonal antibodies used were mAb 62, a monoclonal antibody directed against human Platelet-Endothelial Cell Adhesion Molecule-1 (PECAM) [11], [14] and MEC-13.3 (BD Biosciences, San Jose, CA) towards murine PECAM. Anti-PECAM/SOD conjugates were prepared via amino-chemistry as described earlier [8]. Tumor necrosis factor (TNF) and IL-1β were from BD Biosciences (Bedford, MA).

### Cell Culture and Treatment

Human umbilical endothelial cells (HUVEC; Lonza Walkersville, Walkersville, MD) were maintained in EGM-2 BulletKit medium (Lonza) with 10% fetal bovine serum supplemented with 100 U/ml penicillin and 100 µg/ml streptomycin (GIBCO) in Falcon tissue culture flasks (BD Biosciences, San Jose, CA) coated with 1% gelatin. For activation cells were starved overnight in EBM-2 with 0.5% fetal bovine serum and TNF, IL-1β or poly(I:C) were added to cells for 4–5 h. Lipopolysaccharide (LPS) was added to cells in complete EGM-2 medium.

### Cell Transduction

HUVEC were transduced with adenoviruses Ad-CMV-Luciferase, Ad-NFKb-Luciferase, Ad-SOD1 or Ad-Catalase (Vector Biolabs, Philadelphia, PA) by exposing cells with indicated titer (and MOI) for 2 h. HUVEC were first plated in complete HUVEC EBM medium (10% FBS, EBM and additives; Clonetics) and allowed to grow for 3 days until confluence culture. Then cells were trypsinized, harvested and plated on 96 well culture dishes for luciferase experiments (on 24 well culture dishes in case of transfection with catalase- or SOD-containing viruses) at low density (1∶4 by square area) for 16 h. Cells were exposed to increasing concentrations of adenoviruses for 2 h, washed and incubated for 24 h with fresh medium. In experiments with luciferase-containing adenoviruses cells were further pre-treated with Ab/SOD followed by activation with TNF for another 4 h before luciferase activity assay. Preliminary studies showed dose-dependent increasing luciferase expression by TNF-treated cells with the increase of Ad-NFκB-Luc dose. Optimal vector concentration was chosen as 200 MOI (2×10^7^ PFU/ml) and used for SOD targeting experiments. Cells were preincubated for 1 h with Ab/SOD and treated with 10 ng/ml TNF for 4 h. Transfection was done next day for 2 h in same medium, medium was refreshed and cells were allowed to grow for 24 h before experimental treatment. Ad-CMV-Luciferase was used as a positive control in preliminary experiments. Luciferase activity was measured using RapidReporter Gaussia Luciferase Assay (Active Motif, Carlsbad, CA). Luciferase protein was assayed by Western blotting.

### Western Blotting

Cells in 24 well culture dishes (app. 100,000 cells per well) were washed twice with phosphate-buffered saline (PBS) and lysed in 100 µl of sample buffer for sodium dodecyl sulfate polyacrylamide gel electrophoresis. Cell proteins were subjected to 4–15% gradient gel. Gels were transferred to PVDF membrane (Millipore) and the membrane was blocked with 3% nonfat dry milk in TBS-T (100 mM Tris (pH 7.5), 150 mM NaCl, 0.1% Tween 20) for 1 h followed by incubations with primary and secondary antibodies in the blocking solution. The blot was detected using ECL reagents (GE Healthcare). In experiments with antioxidant enzyme transfection cells were activated with cytokines TNF, IL-1β or with TLR4 agonist LPS for 4 h or with TLR3 agonist poly(I:C) for 5 h.

### RNA Extraction and Real-time Quantitative Reverse-transcriptase PCR

Cells were grown on 24 well culture dishes. Total RNA was extracted using RNeasy Mini Kit (Qiagen, Hilden, Germany) and cDNA was synthesized using Transcriptor First Strand cDNA Synthesis Kit (Roche) in accordance to manufacturer’s recommendations. Levels of mRNA of human VCAM-1 and actin were measured by quantitative real-time RT-PCR using gene-specific primers (LightCycler, Roche) with SYBR green. Primers for actin were from [15] and VCAM-1 from [16]. Reactions were incubated at 95°C at 5 min before thermal cycling at 95°C for 15 s, 60°C for 5 s, and 72°C for 10 s. Samples were prepared in triplicates and transcript expression was calculated by comparing the number of thermal cycles that were necessary to produce threshold amounts of product as described [17] and normalized by β-actin.

### Catalase and SOD Activities

Catalase and SOD activities were measured in lysates of control and transfected cells. Catalase activity was assayed by H_2_O_2_ degradation. Cell lysate was added to working buffer contained of 3% H_2_O_2_ and 5 mM sodium phosphate buffer, pH 7.0. Degradation of H_2_O_2_ was detected at 242 nm using UV-VIS spectrophotometer Cary 50 Bio (Varian, Palo Alto, CA) and calculated as ΔA/min/mg of protein. SOD activity was measured using ferricytochrome assay [18]. Xanthine and xanthine oxidase was used as a source of superoxide anion and cytochrome c as the indicating scavenger of the radical competing with SOD. Working solution contained of 50 mM phosphate buffer (pH 7.8), 0.1 EDTA, 50 µM xanthine, 20 µM cytochrome c (600 µl) and 10 µl of cell lysate. Reaction was initiated by the addition of 10 µl of 0.2 U/ml xanthine oxidase and the absorbance was monitored at 550 nm using Cary 50 spectrophotometer. One unit of SOD activity is defined as the amount of cell lysate, which inhibits the rate of cytochrome c reduction by 50%.

### NF-κB Nuclear Translocation

Cells were treated with TNF for 15 min. Cellular nuclear fraction was isolated using NE-PER® Nuclear and Cytoplasmic Extraction Reagents (Pierce). Activity of NF-κB in the nuclear extract was measured by TransAM NFkB p65 DNA-binding ELISA in accordance with manufacturer’s recommendations (Active Motif, Carlsbad, CA).

### IκBα Phosphorylation

Cells were grown in 6 well culture dishes (4 wells per condition), treated with 10 µM proteasome inhibitor MG132 and Halt™ Phosphatase Inhibitor Cocktail (Pierce) for 30 min, stimulated with TNF for 5 min. After TNF treatment cells were scrapped, centrifuged and lyzed for Western blotting. IκBα phosphorylation was detected using rabbit anti-IκBα antibodies (Santa-Cruz) for loading control and rabbit anti-pS^32,36^-IκBα antibodies (Abcam) for level of protein phosphorylation.

### Intravital Microscopy of Leukocyte-endothelial Interactions

Intravital microscopy for *in vivo* leukocyte adhesion was performed on 8 week old male C57BL/6 mice (20–25 g). These *in vivo* experiments were conducted in strict accordance with the recommendations in the Guide for the Care and Use of Laboratory Animals of the National Institutes of Health and approved by the Institutional Animal Care and Use Committee at Temple University (Permit Number: 3415). All surgery was performed under ketamine/xylazine anesthesia, and all efforts were made to minimize stress and discomfort. Intravital microscopy was performed on animals underwent craniotomy and cranial window implantation after a recovery period of at least 4 days as described earlier [19], [20]. For evaluation of leukocyte rolling and adhesion, cells were stained *in vivo* by an i.p. injection of 200 µl of a 0.05% Rhodamine 6G. The conjugates were administered i.v. 30 min before i.p. LPS (from *E. coli* 0127:B8) injection. Intravital imaging was performed 4 h post LPS injection. Observation of surface cerebral vessels though cranial window was performed with a Stereo Discovery V20 epifluorescence microscope (Carl Zeiss Microimaging Inc., Thornwood, NY) as described [20]. A 30-s video (at 16–20 frame/s) was captured by digital high speed recorder Axiovision module and analyzed using the Imaris imaging software (Bitplane AG, Switzerland).

### Cerebral Reperfusion Model

Transient middle cerebral artery occlusion (MCAO) model (30 min of ischemia followed by 48 h of reperfusion) was performed on mice. Briefly, the left common and internal carotid arteries were ligated and the external carotid artery was isolated and incised. Silicon-covered nylon filament (Doccol) was introduced into the external carotid artery with MCAO with subsequent reperfusion and advanced to the middle cerebral artery for 30 min. as described elsewhere [21], [22]. All procedures were performed in strict accordance with the recommendations in the Guide for the Care and Use of Laboratory Animals of the National Institutes of Health and approved by the Massachusetts General Hospital Subcommittee on Research and Animal Care (Permit Number: 2003N000297). Surgery was performed under isoflurane anesthesia, and all efforts were made to minimize suffering. For measurements of the infarct volume brain tissue was cut into 2-mm-thick coronal blocks, immersed in 2,3,5-triphenyltetrazolium chloride for 12 h. The area of infarction in each section was expressed as a fraction of the non-ischemic part of ipsilateral hemisphere (indirect volume of infarct) as described elsewhere [22].

### Endotoxemia Model in Mice

This study was performed on 6–8 week old male C57BL/6 mice (20–25 g) and was carried out in strict accordance with the recommendations in the Guide for the Care and Use of Laboratory Animals of the National Institutes of Health. The protocol was approved by the Committee on the Ethics of Animal Experiments of the University of Pennsylvania (Permit Number: 803375). All surgery was performed under ketamine/xylazine anesthesia, and all efforts were made to minimize suffering. Conjugates or non-conjugated antioxidant enzymes (150 µg of SOD) were injected 30 min prior LPS (from *E. coli* O55:B5, 200 µg/kg) via tail vein [8]. In 5 hours after LPS challenge lungs were perfused and harvested. VCAM-1 expression level was assayed by Western blot. Plasma TNF and MIP2 level were measured by ELISA using Mouse TNF-α and Mouse CXCL2/MIP-2 Quantikine kits (R&D Systems, Minneapolis, MN).

### Statistical Analyses

Statistical significance was estimated by Student t-test.

## Results

### Protective Effects of Ab/SOD in Animal Models of Vascular Inflammation

Our initial study documented that Ab/SOD inhibits cytokine-induced endothelial expression of inducible adhesion molecule VCAM *in vitro* and *in vivo* [8]. VCAM supports leukocyte adhesion to endothelium at sites of inflammation, and, therefore, by inhibiting this and other pro-inflammatory pathways, Ab/SOD may suppress inflammation [5]. In order to appraise the physiological and potential therapeutic effect of this targeted antioxidant intervention, we tested it in models of acute vascular inflammation.

**Figure 1:**
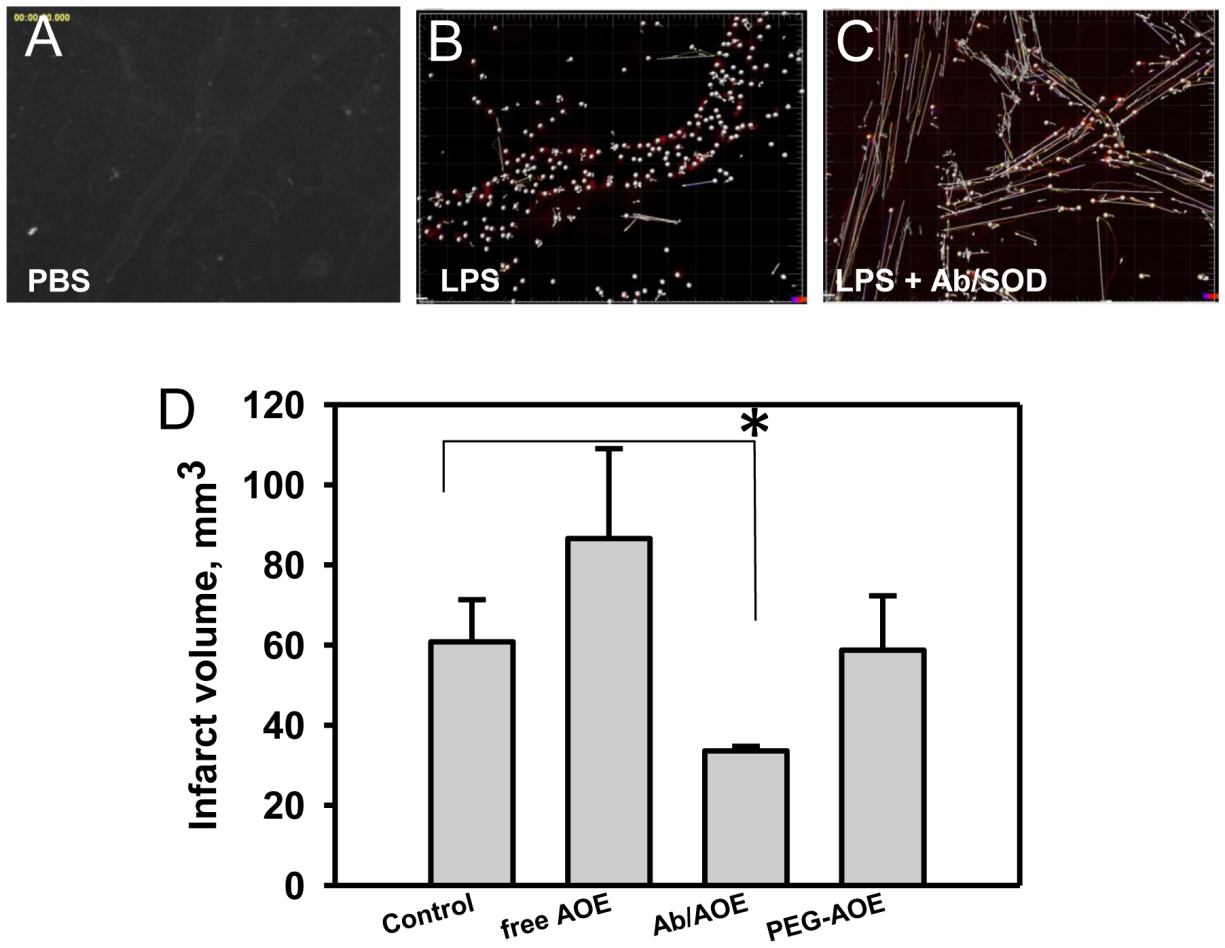
Protective effects of PECAM-directed Ab/AOE targeting *in vivo*. (A–C). SOD targeting inhibits leukocyte adhesion to surface cerebral vessels in an *in vivo* model of inflammation. Mice were intravenously injected with PBS (A), LPS (B) or anti-PECAM/SOD and LPS (C). Labeled leukocytes interaction with endothelium of cerebral vessels was monitored by intravital microscopy through an implanted cranial window at 4 h after the injection. Observation of labeled leukocyte rolling was performed during a 30 s (16–20 frames/s) time-series acquisition. The spots denote the identified leukocytes by the automated particle counting function provided the image analysis. Displacement vectors are shown as tracks to indicate whether the cell is attached (no vector) or rolling (vectors of various lengths) during the image acquisition time interval. (D). Systemic administration of antioxidant enzymes (AOE) SOD and catalase conjugated with PECAM antibody (Ab/AOE) alleviates brain infarction in the filament model of MCAO. * P<0.05, n = 7 (control), 3 (free AOE), 5 (Ab/AOE), and 3 (PEG-AOE).

First, in a model of endotoxin encephalitis, mice with implanted cranial window for observation of surface cerebral vessels were injected with LPS [19]. This caused a firm adhesion of leukocytes to the vessel wall with just a few rolling leukocytes (Fig. 1A and B). The images were processed for automated particle counting (identifying the location of the leukocytes) showing also vector/track projections to enable the distinction between rolling and attached cells. Ab/SOD attenuated endotoxin-induced leukocyte adhesion in the cerebral vessels: note the drastic decrease of adherent leukocytes and increased level of rolling cells unable to anchor to the endothelium (Fig. 1C). Therefore, quenching endothelial superoxide by Ab/SOD does provide anti-inflammatory effects *in vivo* consistent with the observed earlier suppression of endothelial cell adhesion molecules [8].

Based on these encouraging results, we tested whether targeting enzymes quenching endothelial ROS provides tangible therapeutic benefits in an animal model that resembles ischemic stroke in humans, namely, a mouse model of MCAO by filament followed by reperfusion [21], [22]. Injection of antioxidant enzymes (AOE, catalase and SOD) or PEG-conjugated AOE that have prolonged circulation and enhanced systemic bioavailability had no effect on the brain infarction size (Fig. 1D). In contrast Ab/AOE significantly attenuated cerebral injury in this model (Fig. 1D).

### Ab/SOD Inhibits NF-κB Pathway Activation by Cytokines

Next we studied the mechanism of the protective effects. Our previous work established that Ab/SOD targeted to PECAM binds to endothelial cells, delivers SOD into endosomes and inhibits VCAM expression induced by cytokines or TLR agonists, whereas Ab/catalase or untargeted enzymes including PEG-SOD did not have this effect [8]. To elucidate the mechanism of action of Ab/SOD, we tested whether the targeted SOD can directly affect the NF-κB signal transduction pathway, known to play the central role in endothelial activation by cytokines [23].

**Figure 2:**
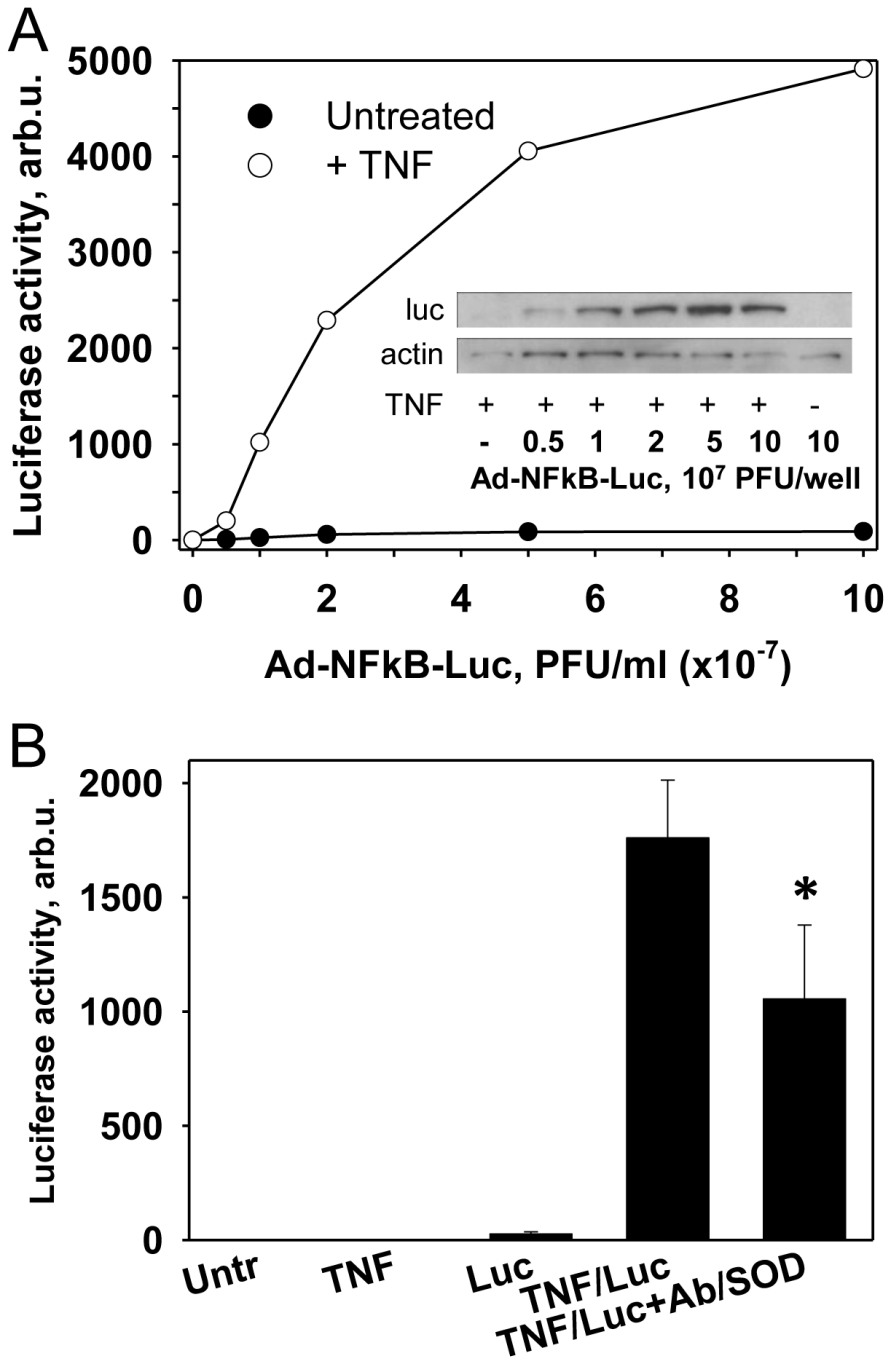
PECAM-directed Ab/SOD delivery to endothelium inhibits TNF-activated NF-κB signaling. (A). Expression of NFkB-dependent luciferase by TNF-activated cells. HUVEC were transfected for 2 h with Ad-NFkB-Luc at 1×10^7^ PFU/ml (100 MOI), vector was washed out and cells were incubated for 16 h followed by activation with 10 ng/ml TNF for 4 h. Cells were lysed. Luciferase expression was assessed by Western blotting (inset), luciferase activity was measured by Luciferase activity assay (Promega). (B). Effects of Ab/SOD targeting. HUVEC were transfected with Ad-NFkB-Luc at 1×10^7^ PFU/ml (100 MOI) for 2 h, vector was washed out and cells were incubated for 16 h. Transfected cells were treated with Ab/SOD targeted to PECAM for 1 h and activated with 10 ng/ml TNF for 4 h. Cells were lysed and luciferase activity was measured by Luciferase activity assay (Promega). Non-transfected cells (without or with TNF activation) did not show luciferase activity. Mean±SEM are shown, * P<0.05, n = 4.

For this purpose we used HUVEC transfected with luciferase reporter Ad-NFκB-Luc driven by NF-κB response element, showing dose dependent response to TNF (Fig. 2A). In this model system Ab/SOD inhibited TNF-induced NF-κB activation by 40% (Fig. 2B). To confirm this outcome, we tested the effect of Ab/SOD on NF-κB nuclear translocation. Using ELISA for the detection of p65 subunit of NF-κB we found that Ab/SOD, but not untargeted free SOD significantly decreased the TNF-enhanced nuclear p65 level by ∼30% (not shown).

**Figure 3:**
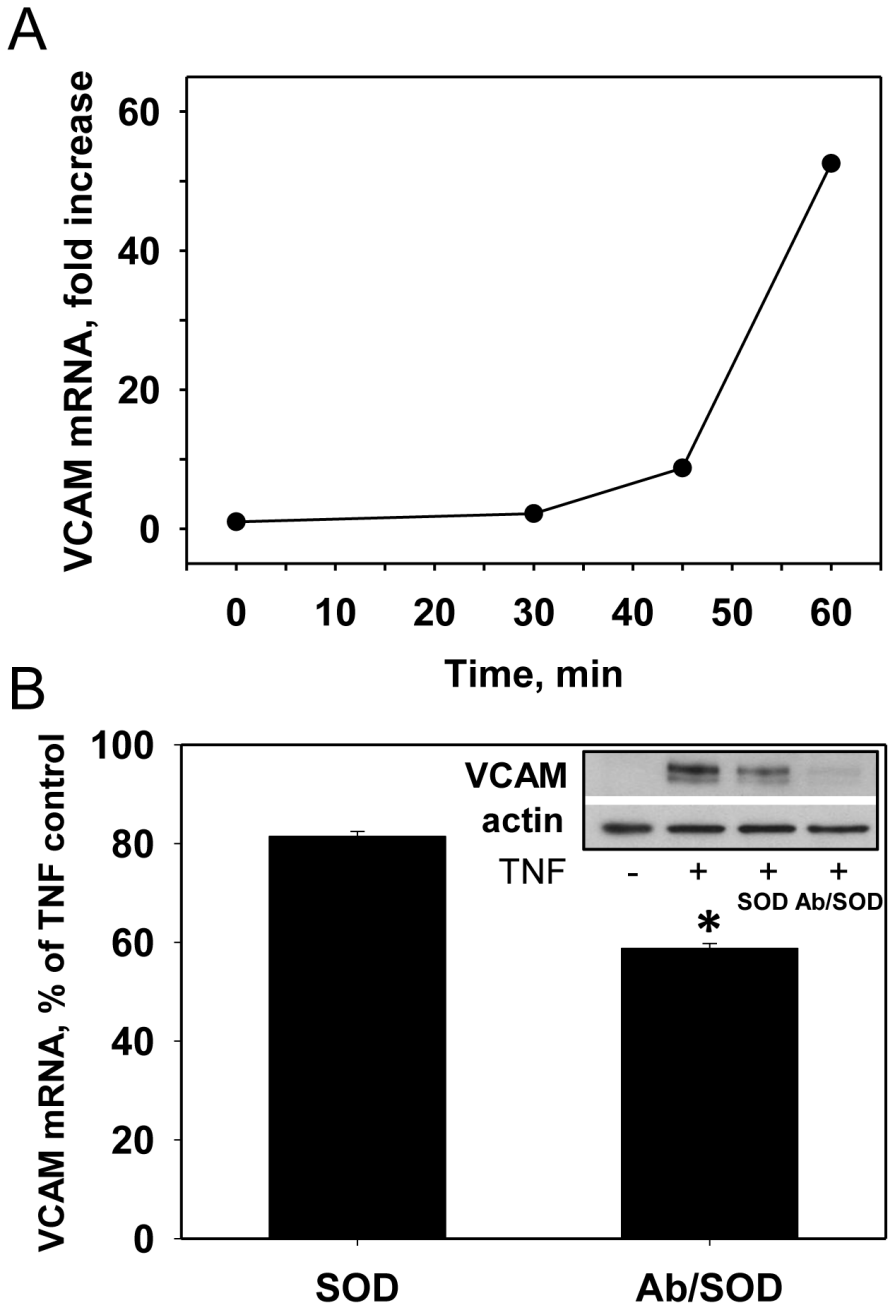
PECAM-directed Ab/SOD targeting to endothelial cells inhibits TNF-induced synthesis of VCAM. (A). Kinetics of synthesis of VCAM after endothelial cells induction by 10 ng/ml TNF as measured by qPCR. (B). Effects of Ab/SOD targeting. Cells were pretreated with anti-PECAM/SOD or untargeted SOD for 1 h and activated with 10 ng/ml TNF. Level of VCAM mRNA was measured by qPCR in 1 h. *p<0.05 vs. control. (Inset), representative Western blot analysis of VCAM protein after 5 h.

Therefore, Ab/SOD inhibits cytokine-induced NF-κB activation in endothelial cells, which can suppresses its downstream effects. This notion was supported by the effects of Ab/SOD on VCAM mRNA level measured by qPCR. Preliminary data on the kinetics of VCAM mRNA expression in response to TNF showed that in 1 h after addition of the cytokine the level of VCAM mRNA was augmented 53-fold (Fig. 3A). Further we observed that in 1 h after TNF exposure Ab/SOD, but not untargeted free SOD significantly decreased the level of VCAM mRNA by 40% (Fig. 3B). Consistent with previous study [8] protein level of VCAM was profoundly attenuated by cell treatment with Ab/SOD (Fig. 3B, inset). It is not clear, how this relatively fair decrease in VCAM mRNA results in a profound suppression observed at the protein level. A change in turnover, shedding, degradation or misfolding of the transcribed protein, either constitutive or aberrant due to altered redox state, seems as plausible explanations.

### Endothelial Activation by Diverse Pro-inflammatory Agonists is Mediated by Intracellular Superoxide

These results square well with previous findings that Ab/SOD inhibited VCAM synthesis induced by cytokines (TNF and IL-1β) or TLR agonists (LPS and poly(I:C)) [8]. These agonists act upon different cognate receptors that, upon ligand engagement, undergo endocytosis via either caveolar (TNF, IL-1β and LPS) or clathrin-mediated (poly(I:C)) pathways and concomitantly activate NADPH oxidase in endosomal membrane, which fluxes ROS into the endosomal lumen (see Discussion for details). Quenching superoxide in endosomes attained by Ab/SOD or blocking the anion channel ClC3 responsible for the transfer of superoxide from endosomes to cytosol inhibited cell activation [8].

**Figure 4:**
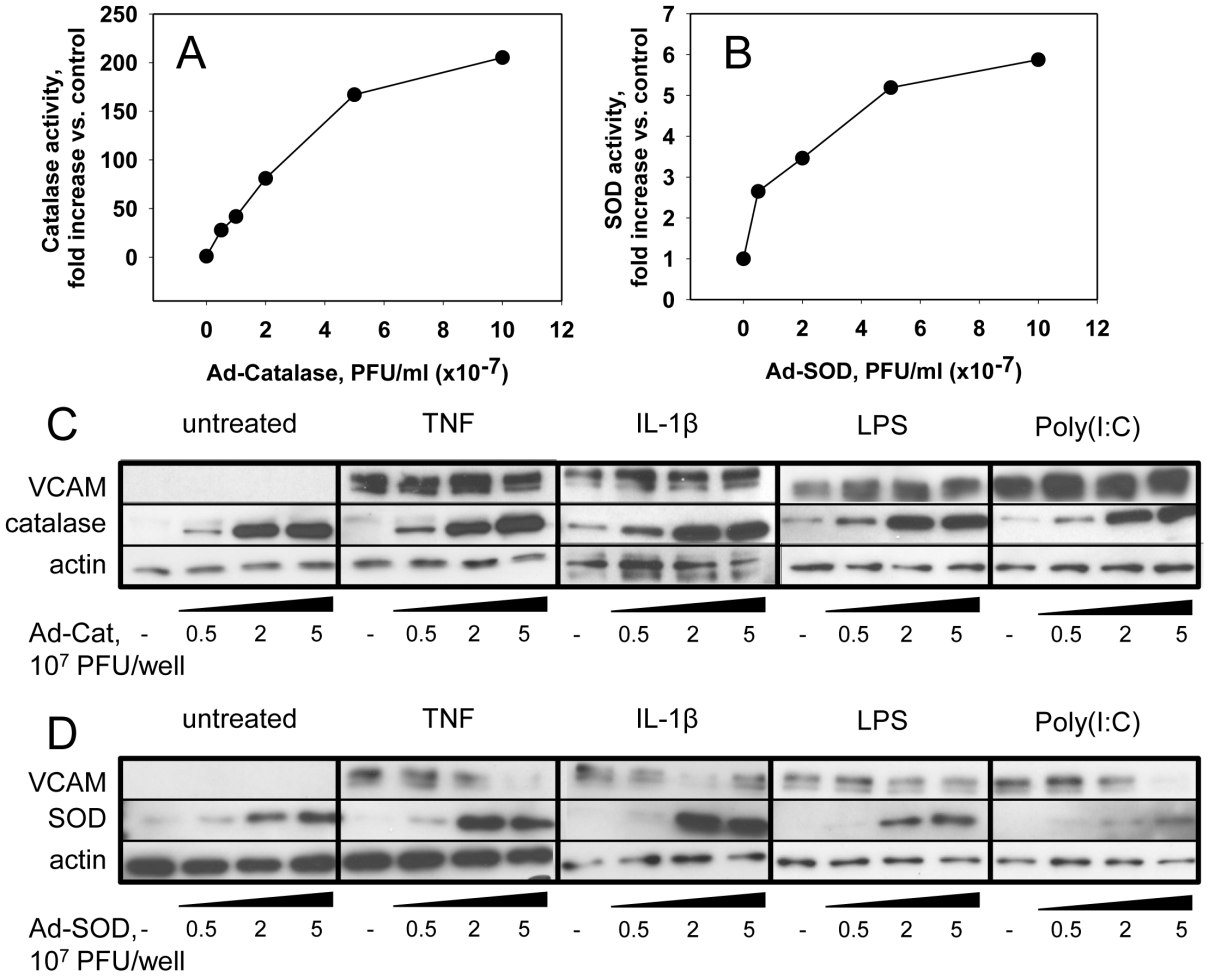
SOD1, but not catalase overexpression in endothelial cells inhibits VCAM expression induced by cytokines or TLR agonists. A–B. HUVEC were transfected with increasing doses (0–10×10^7^ PFU/ml; 0–1000 MOI) of either Ad-Cat or Ad-SOD1 adenoviral vectors. Enzyme activities of catalase (A) or SOD (B) were measured in corresponding cell lysate to test the level of the antioxidant enzyme expressions. C–D. Effects of catalase and SOD1 overexpression on cellular responsiveness to cytokines and TLR agonists were assayed by Western blotting of VCAM. Cells transfected with increasing doses (0–5×10^7^ PFU/ml; 0–500 MOI) of Ad-Cat (C) or Ad-SOD1 (D) were activated with 10 ng/ml TNF, 10 ng/ml Il-1β, 0.5 µg/ml TLR4 agonist LPS, or 20 µg/ml TLR3 agonist poly(I:C). VCAM expression was detected in 4 h for TNF, IL-1β and LPS treatment or in 5 h for poly(I:C) treatment.

To understand whether superoxide transported from endosomes to cytosol acts as the direct signaling molecule or rather serves as precursor of hydrogen peroxide acting upon cognate cytosolic sensors, we transfected cells with SOD1 or catalase using adenoviral vectors. This approach provided a dose-dependent elevation of the enzymatic activity in cell lysates that reached maximal levels of 200-fold and 6-fold increase over the basal level for catalase and SOD, respectively (Fig. 4, A and B). Further we demonstrated that expression of SOD, but not catalase inhibited VCAM expression induced by TNF, IL-1β, LPS or poly(I:C) (Fig. 4, C and D). Moreover, the inhibitory effects of SOD clearly depended on its expression level (Fig. 4D).

### The Combined Effect of Ab/SOD and Exogenous NO Donors

Nitric oxide is known to inhibit NF-κB pathway in endothelial cells [24], [25]. Based on the fact that the mutual influences of NO and ROS are of paramount importance in vascular biology, inflammation and vessel tone control [26], [27], we wanted to study the interrelationships between Ab/SOD and NO donors in the context of cytokine activation of endothelium.

**Figure 5:**
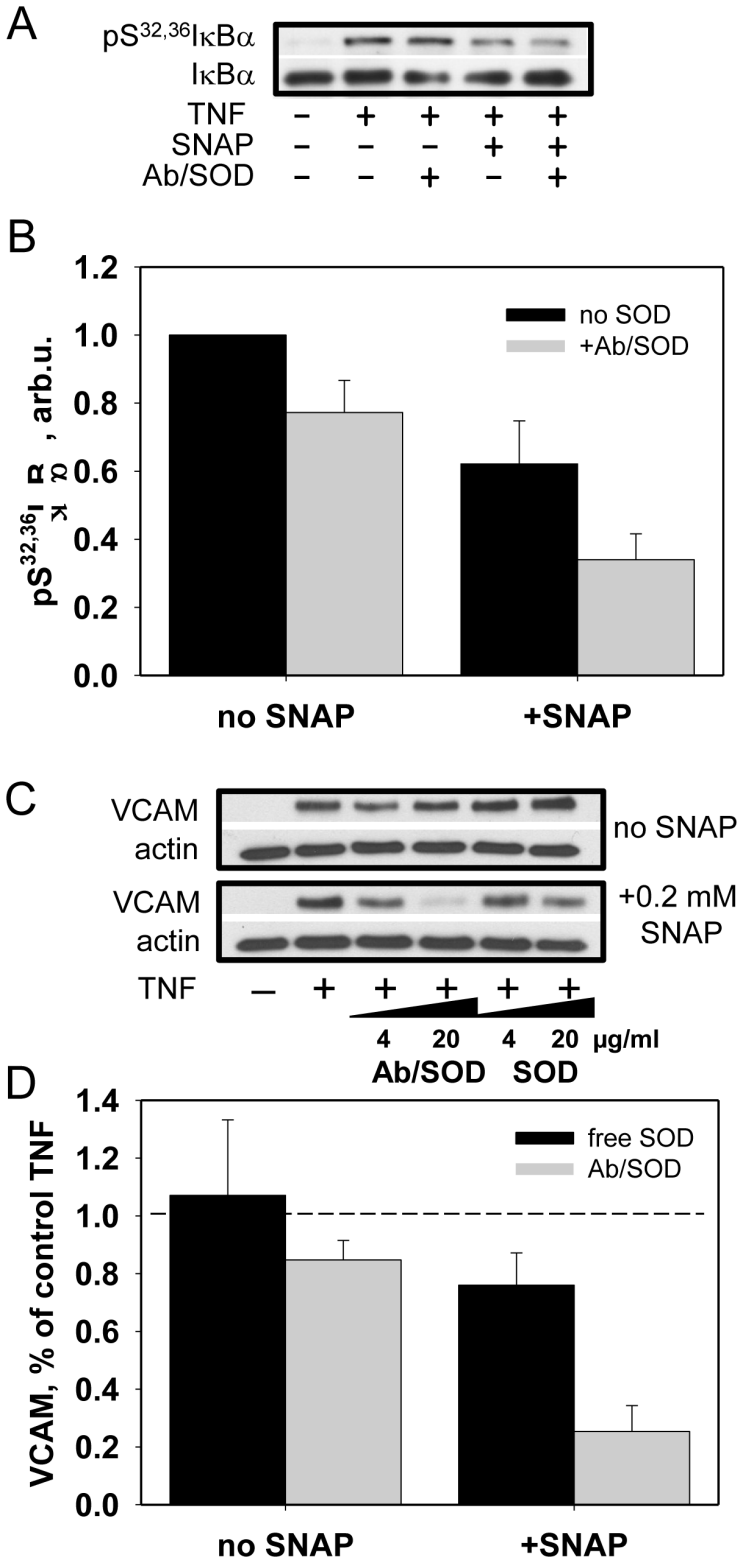
Ab/SOD potentiates anti-inflammatory effect of NO donor SNAP *in vitro*. (A–B). Western blotting analysis of IκBα phosphorylation. HUVEC were treated with SNAP (0.2 mM), washed, treated with anti-PECAM/SOD (20 µg/ml SOD) for 1 h followed by activation with TNF (10 ng/ml) for 10 min. Western blotting (A) and level of phosphorylated IκBα normalized by total IκBα (B) are shown. (C–D). VCAM expression by HUVEC co-treated with SNAP and anti-PECAM/SOD. Experimental conditions described above. Cells were activated with TNF for 4 h. Western blotting (C) and VCAM level normalized by actin (D) are shown. Representative images from at least three experiments are shown.

First, we refined the dose dependence of inhibition by NO-donor SNAP of the TNF-induced NF-κB activation and VCAM expression in endothelial cells (Fig. S1). Therefore, we choose the doses of Ab/SOD and SNAP (20 µg/ml and 0.2 mM, respectively), which individually caused rather marginal inhibition of TNF-induced endothelial activation. Using these rate-limiting doses we found that combined treatment of TNF-activated cells with Ab/SOD and SNAP lead to significant decrease of IκBα phosphorylation (Fig. 5, A and B) and complete inhibition of VCAM expression (Fig. 5, C and D). In contrast, non-targeted SOD did not potentiate the anti-inflammatory effect of SNAP (Fig. 5, C and D); which implies that it is interception of intracellular superoxide poorly accessible to free SOD in the medium plays the key role in augmentation of effects NO.

**Figure 6:**
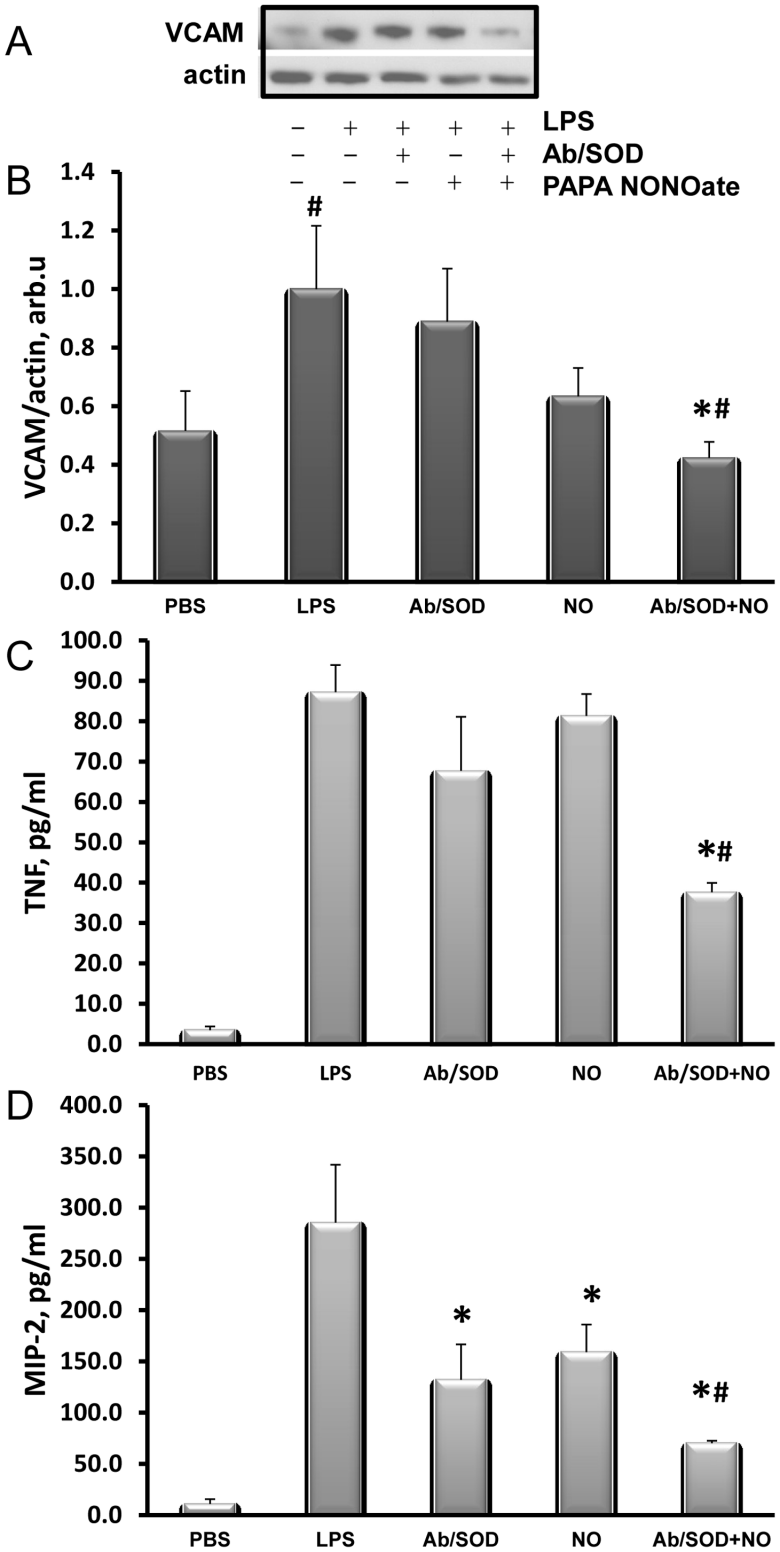
Ab/SOD potentiates anti-inflammatory effects of NO donor in LPS-challenged mice. Animals were injected intravenously (in tail vein) with anti-PECAM/SOD (25 µg/mouse), PAPA NONOate (prepared immediately before use, 0.1 mM final concentration, based on blood volume of 7.5% of body weight) or their combination. LPS (200 µg/kg) was injected same way in 30 min. Lung tissue and plasma were harvested in 5 h. Lung VCAM level was assayed by Western blotting: (A), representative image; (B), Western blotting analysis. Plasma TNF and MIP-2 concentrations were measured by corresponding ELISA (C and D, respectively). Means ± SEM are shown; n≥3, * p≤0.05 vs. LPS-treated group; # p≤0.05 vs. LPS+NO group.

The results suggest that two potential therapeutics exerting distinct activities (targeted SOD and systemic NO donors) can act in concert to alleviate vascular oxidative stress and inflammation. To test this intriguing scenario *in vivo*, we used mouse model of endotoxin challenge, leading to pro-inflammatory elevation of VCAM-1 in the pulmonary vasculature [8]. Analysis of the lung homogenate revealed that combined treatment with Ab/SOD and NO-donor PAPA NONOate at rate-limiting doses that failed individually provided nearly complete suppression of LPS-induced VCAM expression in the pulmonary vasculature (Fig. 6, A and B).

Furthermore, analysis of plasma TNF and MIP-2 levels revealed a very similar outcome: used in a concert, NO-donor and Ab/SOD afforded significantly more potent suppression of cytokine level in blood in response to endotoxemia (Fig. 6, C and D, respectively). Thus, NO donor and Ab/SOD attenuated both local (lung VCAM) and systemic (plasma cytokines) inflammatory markers.

## Discussion

Targeted delivery of antioxidant enzymes (e.g., catalase and SOD) to endothelial cells affords multifaceted protective effects in diverse pathological settings associated with vascular oxidative stress [13], [28]. Affinity ligands used for this purpose include antibodies and antibody fragments binding to PECAM [9], [10], [29], [30], ICAM-1 [31], [32] and angiotensin-converting enzyme [33], as well as peptides binding to glycocalyx [34]. Ab/catalase is protective against lung injury caused by ischemia-reperfusion [33], [35], [36], whereas Ab/SOD attenuates angiotensin-II caused vasoconstriction [35], as well as cytokine-induced inflammation [8] and trans-endothelial permeability [37]. Such a functional selectivity of Ab/catalase vs Ab/SOD, taken together with lack of effects of non-targeted enzymes including PEG-conjugated enzymes, imply that in order to achieve protective effects it is necessary to quench ROS specifically in the target cells, i.e., endothelium. Results of the present study recapitulate this paradigm and shed light on the mechanisms and translational potential of targeted interception of superoxide in the endothelial cells.

Pro-inflammatory endothelial activation by agonists involves distinct series of receptors and endocytic pathways. Cytokine receptors IL-1R1 [38] and TNFR1 [39], [40], [41] as well as TLR4 [42] act predominantly via caveolae-mediated endocytosis [43], whereas activation of intracellular receptors such as TLR3 depends on clathrin-mediated endocytosis of dsRNA [44]. Earlier it was demonstrated that TNF-mediated activation of NFκB pathway may be inhibited by transient overexpression of cytosolic SOD1 [45], [46] or mitochondrial SOD2 [47], [48], but not catalase [49], [50]. Stable expression of catalase also inhibited TNF-induced NF-κB [51], due to probably more general alteration of cell functions [49]. In the present study expression of SOD, but not catalase in the cytosol, inhibited signal transduction from cytokine receptors and TLRs to NF-κB path. This result implicates superoxide transferred into the cytosol from endosomes as a direct signaling molecule, and warrants further studies to reveal a cytosolic target of signaling superoxide. Thus, our results help to resolve the controversy, affirming that superoxide, but not H_2_O_2_ mediates NF-κB activation and VCAM expression caused by the array of pro-inflammatory agents acting via distinct receptors and endosomes (Figure 4). From the therapeutic perspective, our results imply that targeted delivery of SOD into endothelial cells intercepts the ROS in signaling endosomes prior to its transfer to the cytosol, thereby providing anti-inflammatory effect.

Many processes in vascular physiology and pathology including ischemia and inflammation involve multifaceted interactions between ROS and nitrogen species in endothelial cells [4], [52]. In particular, superoxide rapidly reacts with nitric oxide, forming a strong oxidant ONOO^−^ and abolishing effects of NO [26]. Previously we have shown that Ab/SOD normalizes vasoregulation in angiotensin II-treated mice by preserving NO endogenously produced by endothelial NOS [35]. Data obtained in the present study indicate that Ab/SOD also augments anti-inflammatory effects of exogenous NO donors, both in cell culture and in animal model (Fig. 5 and 6). The precise mechanism of the potentiation is still to be elucidated. It’s not clear yet whether SOD effect is due to NO preservation or to inhibition of the signaling in NO-independent manner. It is tempting to postulate that potentiation of the effect of the combination of NO donors with targeted SOD may be used to increase efficacy and reduce therapeutic doses of both drugs. Considering multiple systemic effects of NO including hypotension and platelet inhibition, protective anti-inflammatory effect of combined delivery of SOD and NO donors can be improved, in theory, by targeted co-delivery of both agents. The nanocarriers containing NO-donors have been devised and tested in animal studies [53], [54] and may provide a helpful platform for this goal. Further development of this approach, for example, co-loading SOD and NO-donors in hydrogel nanoparticles decorated with targeting antibodies seems warranted.

Studies in mouse models of acute inflammatory insults in cerebral and pulmonary vasculature confirmed the hypothesis that Ab/SOD affords superior protective effects vs untargeted SOD formulations including long-circulating PEG-SOD (Fig. 1D and [8]). It has been shown recently that pluronic based polymer nanoparticles containing catalase and SOD (“nanozyme”) protect brain against ischemia/reperfusion in a MCAO model in rats [55]. This model, also used in our study, recapitulates many pathological components typical of ischemic stroke in humans and is characterized by endothelial exposure of cell adhesion molecules and leukocyte-mediate inflammatory brain injury [22], [56]. Interestingly, “nanozyme” also provided superior outcomes vs standard PEG-enzymes in chronic models of central nervous system pathology including neurodegenerative conditions [57]. In contrast with Ab/SOD, nanozymes are not targeted to endothelium and the mechanism of their effect can be rather attributed to enhanced permeation into the central nervous system parenchyma, where they may protect neurons [58]. It is tempting to postulate that a combination of targeted delivery of antioxidant enzymes into endothelium and accumulation in the CNS parenchyma attained by Ab/conjugates and nanozymes, respectively, may afford the most effective protection. This scenario is worth experimental testing including side-by-side characterization of the pharmacokinetics and tissue uptake of these prospective therapeutic agents. In any case, mechanisms of their effects need to be further defined in order to support their medical and investigational use.

In conclusion, results presented in this paper affirm the central role of superoxide transferred from endosomes to cytosol in pro-inflammatory activation of endothelium by cytokines and TLR ligands. Our data also indicate that interception of this signal by targeted delivery of Ab/SOD may provide both direct anti-inflammatory effect(s) and augment those of other anti-inflammatory agents such as NO donors. This warrants further elucidation of mechanisms, pharmacokinetics, translational potential and challenges of targeted antioxidants, solo and in combination with other means including NO donors and nanozymes.

## Supporting Information

Figure S1 Inhibition of TNF-induced NF-κB signal transduction by NO donor SNAP. (A). Inhibition of NFkB-dependent luciferase activity in TNF-activated transfected cells by SNAP. HUVEC were transfected for 2 h with Ad-NFkB-Luc at 100 MOI (1×10^7^ PFU/ml), vector was washed out and cells were incubated for 16 h. After that cells were treated with indicated concentration of SNAP for 30 min, washed and exposed to 10 ng/ml TNF for 4 h. Luciferase activity was measured by Luciferase activity assay (Promega). (B–C). Inhibition of VCAM-1 expression. Cells were pre-treated with SNAP for 30 min, washed and activated with TNF (10 ng/ml) for 4 h. VCAM-1 expression was assayed by Western blotting (B) and normalized by actin (C).
(TIF)
