# Supplementary figures and images for: Anti-Inflammatory Effect of Targeted Delivery of SOD to Endothelium: Mechanism, Synergism with NO Donors and Protective Effects In Vitro and In Vivo
Source: PLoS One. 2013 Oct 11;8(10):e77002. doi: 10.1371/journal.pone.0077002 (PMC3795626; doi:10.1371/journal.pone.0077002)

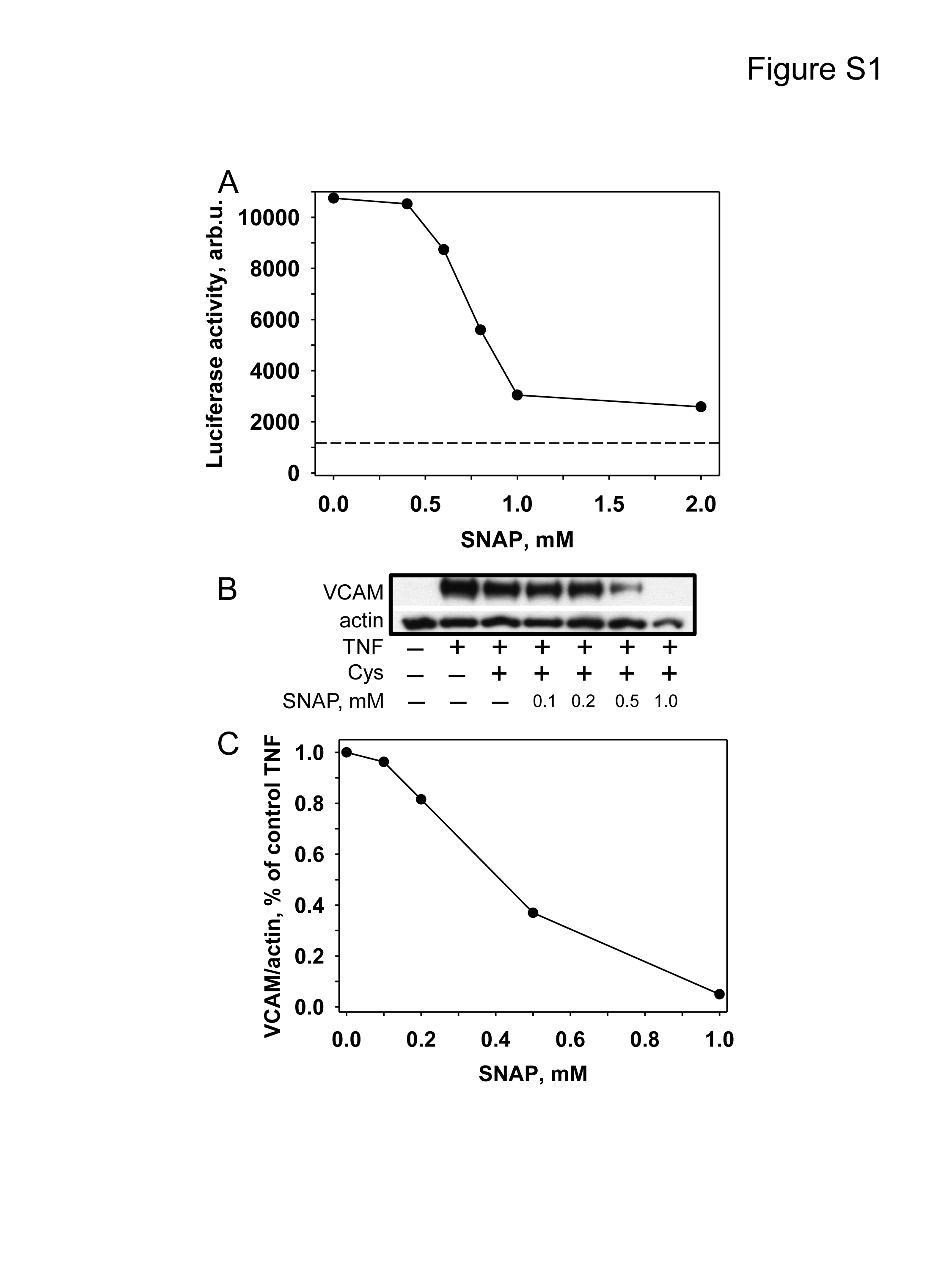

Supplement: Figure S1 — Inhibition of TNF-induced NF-κB signal transduction by NO donor SNAP. (A). Inhibition of NFkB-dependent luciferase activity in TNF-activated transfected cells by SNAP. HUVEC were transfected for 2 h with Ad-NFkB-Luc at 100 MOI (1×10^7^ PFU/ml), vector was washed out and cells were incubated for 16 h. After that cells were treated with indicated concentration of SNAP for 30 min, washed and exposed to 10 ng/ml TNF for 4 h. Luciferase activity was measured by Luciferase activity assay (Promega). (B–C). Inhibition of VCAM-1 expression. Cells were pre-treated with SNAP for 30 min, washed and activated with TNF (10 ng/ml) for 4 h. VCAM-1 expression was assayed by Western blotting (B) and normalized by actin (C). (TIF) [file pone.0077002.s001.tif]
